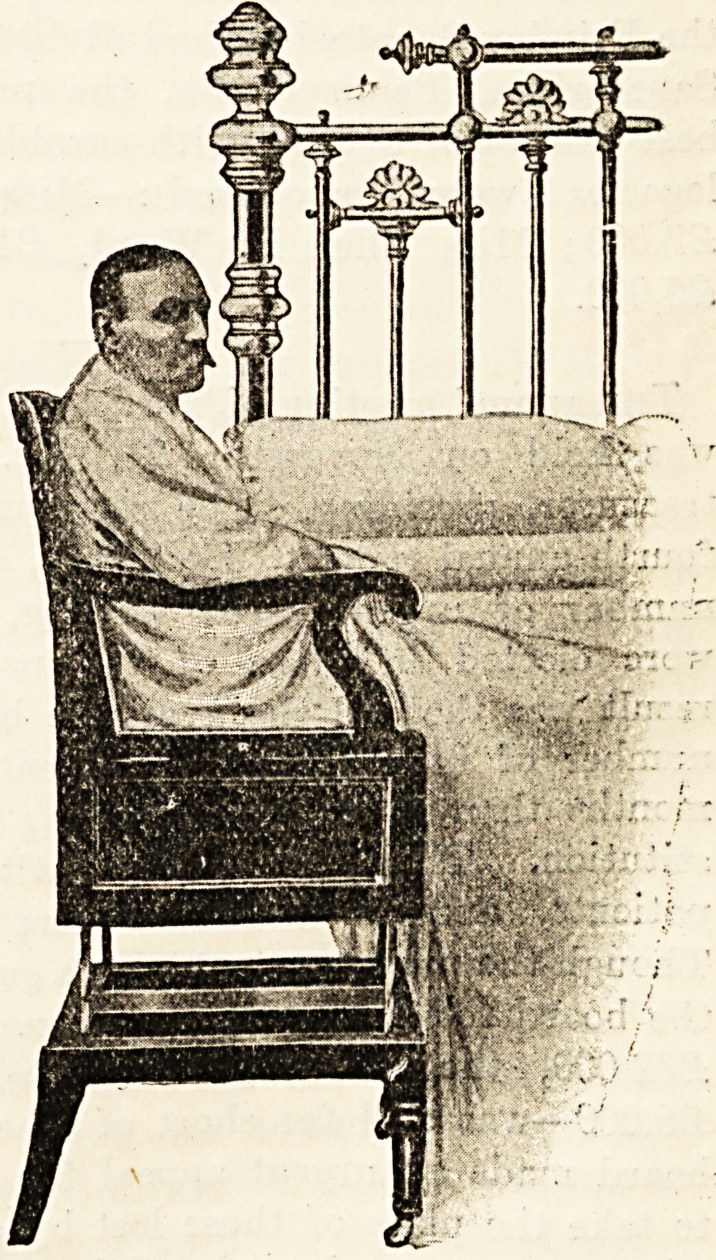# New Appliances and Things Medical

**Published:** 1908-12-05

**Authors:** 


### NEW APPLIANCES AND THINGS MEDICAL.

### A NEW RISING-SEAT COMMODE.

DR. ARMSTEAD, of 30 Queensborough Terrace, Hyde Park, W., writes as follows: In attending a case recently, in which the patient was so averse to the use of the bed-pan that aperient medicines had to be constantly given, it occurred to me that if the commode in common use could be made adjustable to the height of *any* bed, the patient could with very little assistance shift himself on and off it with comparative ease, the difficulty of *lifting* a heavy patient back to bed from the ordinary commode being thus overcome. This suggestion of mine has been skilfully carried out by J. and A. Carter, of 2-6 New Cavendish Street, W., and many other useful additional features added. The arms of this chair turn down, leaving the seat flush with the height of any bed, and a firm, strong footboard is fitted, so that the patient can slide off either side on to the seat and occupy a natural position, or can sit on the commode with the legs remaining in bed. A false caned seat glides over the orifice when the commode is required for use as an ordinary chair. The mechanism of the rising seat is easily operated by nurse or attendant, by whom this commode will be found, I think, of considerable use.

## Figures and Tables

**Figure f1:**
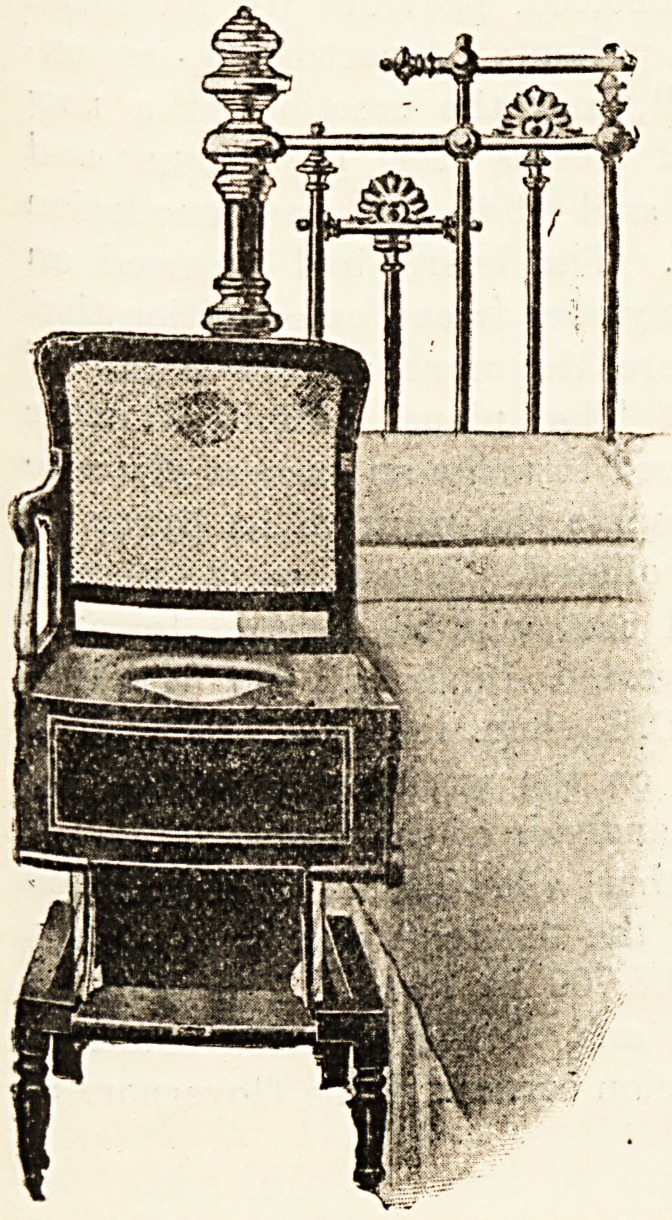


**Figure f2:**